# Biomechanical Effects of Two Types of 3D‐Printed Prosthesis Reconstruction After Single‐Segmental Total En Bloc Spondylectomy: A Finite Element Analysis

**DOI:** 10.1111/os.70336

**Published:** 2026-05-21

**Authors:** Jiasheng Chen, Ben Wang, Yang Luo, Xiang Li, Yanchao Tang, Zihe Li, Hua Zhou, Panpan Hu, Xiaoguang Liu, Zhongjun Liu, Feng Wei

**Affiliations:** ^1^ Department of Orthopaedics Peking University Third Hospital Beijing China; ^2^ Ministry of Education Engineering Research Center of Bone and Joint Precision Medicine Beijing China; ^3^ Beijing Key Laboratory of Spinal Disease Research Beijing China

**Keywords:** 3D‐printed prosthesis, biomechanics, finite element analysis, TES, TMC

## Abstract

**Background:**

Total en bloc spondylectomy (TES) has emerged as an effective surgical intervention for spinal tumor management. The selection of prosthesis for spinal reconstruction significantly influences patient's postoperative outcomes. This study aims to analyze and compare the biomechanical effects of two types of 3D‐printed prostheses and titanium mesh cage (TMC) after TES.

**Methods:**

An intact finite element model (FEM) of L1–L5 segment was developed and validated for simulation. Three L3 TES models were constructed. Model A utilized a 3D‐printed prosthesis with an artificial pedicle, model B employed a stand‐alone 3D‐printed prosthesis, and model C used a TMC. Following parameters were recorded and analyzed to evaluate the biomechanical effects of the three models: (1) the range of motion (ROM), (2) stress of the internal fixation systems, and (3) stress of the L2 inferior endplate and L4 superior endplate.

**Results:**

The ROMs of all three models were significantly restricted in all directions. Compared with TMC, the implantation of 3D printed prosthesis significantly enhanced spinal stability during extension. The ROMs of models A and B were significantly lower than that of model C during extension, decreasing by 15.2% and 36.4%, respectively. The use of 3D‐printed prosthesis for anterior column reconstruction could reduce the stress of prosthesis itself and adjacent endplates. Compared with model C, the maximal decrease in the stress of the endplate of models A and B was 41.5% during flexion and 49.1% during right lateral bending, respectively. In all directions, the stress of the prosthesis was largest in model C, followed by model A and smallest in model B, with statistically significant differences observed.

**Conclusion:**

After single‐segmental TES, 3D‐printed prosthesis with favorable endplate matching could obtain better biomechanical effects, thereby reducing the risk of internal fixation failure and increasing the postoperative spinal stability.

## Graphical abstract

The choice of prosthesis in spinal reconstruction plays an essential role in patients' postoperative outcomes. This study aims to investigate the biomechanical effects of two types of 3D‐printed prosthesis and titanium mesh cage (TMC) after total en bloc spondylectomy. Our research has revealed that compared to TMC, 3D‐printed prostheses with a larger contact area and a lower elastic modulus have demonstrated superior biomechanical properties, effectively reducing the risk of internal fixation failure and enhancing spinal stability.

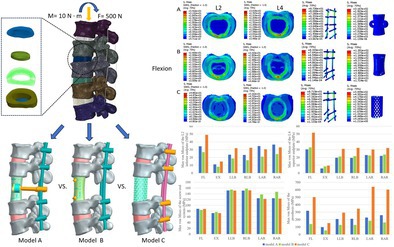

## 1 Introduction

Surgery is the primary treatment for spinal tumors and has been demonstrated to effectively reduce local recurrence, alleviate pain, and preserve or improve neurological function [1]. In recent years, total en bloc spondylectomy (TES) has emerged as an effective treatment for primary and some metastatic spinal tumors and is gaining widespread acceptance among spinal surgeons [2, 3, 4]. The primary objective of TES is to achieve extensive resection of local lesions while preserving tumor boundaries and minimizing the risk of local recurrence, thus prolonging survival and improving quality of life [4, 5, 6, 7]. However, the complete resection of the vertebrae and surrounding ligaments during TES often results in spinal instability. To achieve long‐term stability in spinal reconstruction and preserve postoperative spinal function, posterior fixation combined with anterior vertebral body replacement (VBR) support is essential [8]. This approach can effectively maintain spinal stability, demonstrated by biomechanical experiments [9]. The most widely used VBR technique remains the titanium mesh cage (TMC) combined with autologous and allogeneic bone grafts. However, the TMC's sharp edge, cutting surfaces, and point contact with endplates make it difficult to fix in the adjacent endplates, often leading to stress concentration, prosthesis subsidence, and even instrument failure, which are the major postoperative complications [10]. In recent years, due to the favorable mechanical strength, elastic modulus, and biocompatibility, 3D‐printed prosthesis has gained wide attention in the field of spinal implants [11, 12]. Through computer scanning, porous prosthesis can be precisely customized to match the adjacent endplates and provides an optimal structure for osteoblasts ingrowth. At our center, we have implemented some improvements to traditional 3D printed prostheses, which will be mentioned below.

In 1974, Belytschko et al. [13] first applied finite element analysis (FEA) to biomechanical research in spinal surgery. Since then, FEA has been widely adopted by clinicians for surgical technique selection and prognosis prediction in orthopedic procedures. Compared with traditional experimental methods, FEA enables more comprehensive evaluation of physical parameters and yields more quantitative data. To our knowledge, there is limited literature investigating the biomechanics of 3D‐printed prosthesis for anterior column reconstruction after TES. This study utilizes an L1–L5 finite element model (FEM) to: (i) compare the biomechanics of two 3D‐printed prostheses and TMC after single‐segmental TES; and (ii) inform clinical prosthesis selection based on biomechanical performance.

## 2 Methods

### 2.1 Development of Intact L1‐L5 FE Model

**FIGURE 1:**
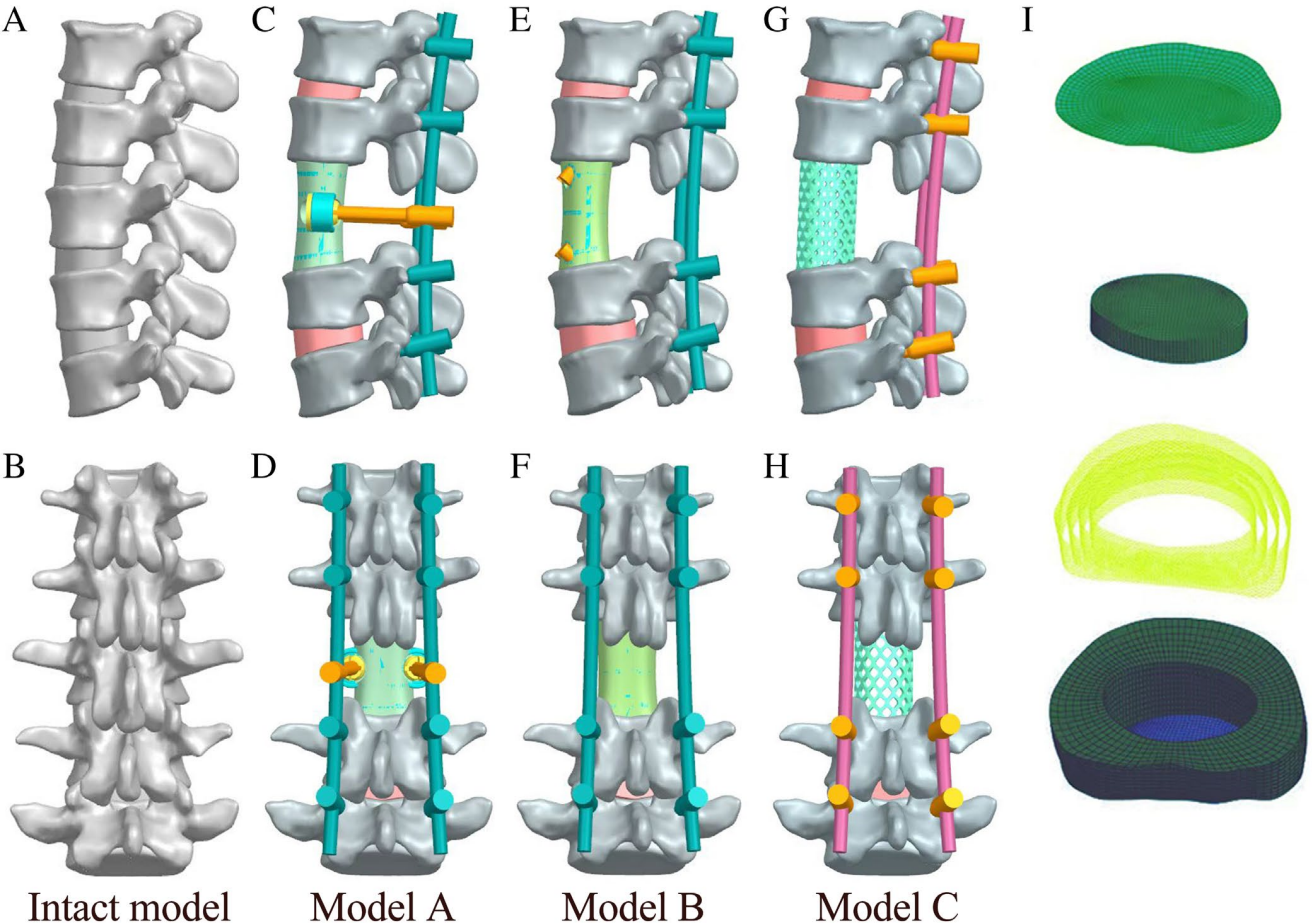
(A, B) Intact geometric model of L1–L5. (C, D) Model A: Model of 3D‐printed prosthesis with artificial pedicle reconstruction after L3 TES. (E, F) Model B: Model of stand‐alone 3D‐printed prosthesis after L3 TES. (G, H) Model C: Model of TMC after L3 TES. (I) the structure of intervertebral disc.

A healthy 35‐year‐old male volunteer with no history of spinal diseases or osteoporosis was selected to develop a normal lumbar FEM. The imaging data in DICOM format of five vertebral bodies and four intervertebral discs between L1 and L5 were obtained with a 64‐slice spiral computed tomography scanner (Siemens, Erlangen, Germany) at 1 mm interlayer spacing. The images were subsequently imported into Mimics V21.0 (Materialize, Leuven, Belgium) to reconstruct an accurate 3D surface model of the L1–L5 region, which was stored as STL format files. Geomagic Studio V12.0 (Geomagic, Cary, NC, USA) was utilized for wrapping, smoothing, surface modeling, and mesh generation. Hypermesh 2017 (Altair Engineering, Troy, MI, USA) was utilized to construct the bone, intervertebral disc, and ligament structures. The intervertebral disc comprised the nucleus pulposus, annulus fibrosus, and endplates, with the nucleus pulposus occupying 30%–40% of the intervertebral volume. The annulus fibrosus consisted of the annulus fibrosus matrix and collagen fibers, which were modeled in three–five layers at an angle of 25°–45° relative to the horizontal surface [14]. The thickness of the endplates was 0.5 mm. Seven ligaments including the anterior longitudinal ligament, posterior longitudinal ligament, ligamentum flavum, interspinous ligament, capsular ligament, intertransverse ligament, and supraspinous ligament were generated for each segment. Two‐node truss elements with no compression stress were utilized to simulate fibers and ligaments. The contact of the facet joint was modeled as frictionless sliding. Abaqus 2020 (Dassault Systèmes Simulia Corp., Providence, RI, USA) was employed for model assembly, material property definition, load application, and FEA [15]. The model was validated based on published FEMs and human spine cadavers (Table 1) [16, 17, 18]. The thicknesses of the cortical bone and facet joint were set to 1 and 0.2 mm, respectively [19, 20]. The intact FEM of L1–L5 was presented in Figure 1.

**TABLE 1:** Material properties of the L1–L5 finite element model.

Material	Element type	Young's modulus (MPa)	Poisson's ratio	Cross‐sectional area (mm^2^)
Vertebrae				
Cortical bone	C3D4	12,000	0.3	
Cancellous bone	C3D4	100	0.2	
Posterior elements	C3D4	3500	0.25	
Cartilage	C3D8R	10	0.4	
Disc				
Nucleus pulposus	C3D8RH	Hyperelastic, Mooney‐Rivlin C^10^ = 0.18, C^01^ = 0.045	0.49	
Annulus fibrosus matrix	C3D8RH	Hyperelastic, Mooney‐Rivlin C^10^ = 0.12, C^01^ = 0.03	0.45	
Fiber	T3D2	360–550	0.30	0.15
Endplate	C3D8R	3000	0.25	
Ligaments				
ALL	T3D2	7.8 (< 12%), 20 (> 12%)	0.3	63.7
PLL	T3D2	10 (< 11%), 20 (> 11%)	0.3	20
LF	T3D2	15 (< 6.2%), 19.5 (> 6.2%)	0.3	40
ISL	T3D2	10 (< 14%), 11.6 (> 14)	0.3	40
CL	T3D2	7.5 (< 25%), 32.9 (> 25%)	0.3	30
ITL	T3D2	10 (< 18%), 58.7 (> 18%)	0.3	1.8
SSL	T3D2	8 (< 20%), 15 (> 20%)	0.3	30
Implants				
Screw‐rod system	C3D4	110,000	0.3	
Titanium mesh cage	C3D4	110,000	0.3	
3D‐printed prosthesis	C3D4	675	0.3	

Abbreviations: ALL, anterior longitudinal ligament; CL, cystic ligament; ISL, interspinous ligament; ITL, intertransverse ligament; LF, ligamentum flavum; PLL, posterior longitudinal ligament; SSL, supraspinous ligament.

### 2.2 Development of Implants and Fixation Models

The posterior fixation system was composed of two rods (5.5 mm in diameter) and eight pedicle screws (6.5 mm in diameter and 45 mm in length), which were designed using SolidWorks (Dassault Systèmes, Paris, France). The pedicle screws were bilaterally inserted into the pedicles of L1, L2, L4, and L5 vertebrae. The pedicle screws were rigidly affixed to the two rods (Figure 1). It was assumed that all pedicle screws were firmly anchored to the vertebral bodies, with no relative motion allowed. The 3D‐printed prosthesis was validated using Geomagic Studio v12.0, with its dimensions precisely determined based on the FEM. The material properties for each component of the model were based on previous literature [16] and were summarized in Table 1. L3 vertebra, along with its associated ligaments and intervertebral discs connecting to adjacent vertebrae, was excised. A Boolean operation was performed to integrate the prosthesis and posterior fixation system, ensuring complete fitting between the prosthesis and the vertebral bodies. Hypermesh 2017 was employed to generate mesh. The intact model was modified to simulate spinal reconstruction with different prostheses. Three postoperative FEMs were ultimately developed. In model A, the VBR consisted of a 3D‐printed prosthesis with artificial pedicle; in model B, the VBR was a stand‐alone 3D‐printed prosthesis; and in model C, the VBR was a TMC.

### 2.3 Boundary Conditions and Outcome Measures

Abaqus 2020 (Dassault Systèmes Simulia Corp., Providence, RI, USA) was utilized to define boundary, apply load condition, and simulate spinal motion. The inferior surface of the L5 vertebra was assumed to be fully constrained, and its substructure was set to the boundary, preventing any displacement or rotation in all directions. Spinal motions were defined in the sagittal, coronal, and transverse planes as flexion‐extension, lateral bending, and axial rotation, respectively. A combined loading condition consisting of an axial preload of 500 N and a pure moment of 10 N·m was applied to the superior surface of the L1 vertebra to simulate six directions: flexion (FL), extension (EX), right/left lateral bending (RLB/LLB), and right/left axial rotation (RAR/LAR) [21, 22]. The peak von Mises stress values (MPa) were calculated and recorded for the prosthesis, internal fixation system, and the endplate adjacent to the prosthesis in each model. Furthermore, the range of motion (ROM) for each model was calculated to assess the biomechanical effects. Statistical analysis was not performed in this FEA due to the single‐subject nature of the model.

## 3 Results

### 3.1 Validation of the FEM

**FIGURE 2:**
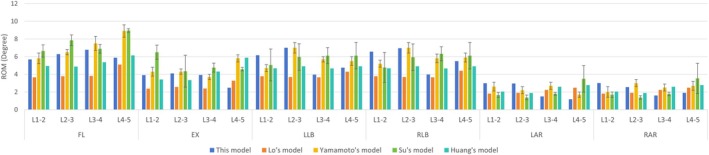
Comparison of the ROM data from the intact L1–L5 model in this study with previously reported experimental data from Yamamoto et al. EX, extension; FL, flexion; LAR, left axial rotation; LLB, left lateral bending; RAR, right axial rotation; RLB, right lateral bending; ROM, Range of motion.

This FEA study measured the ROM of each spinal segment of the intact L1‐L5 model during FL, EX, RLB/LLB, and RAR/LAR, with a 10 N·m torque load applied to the superior surface of the L1 vertebra. These ROM values were consistent with the in vitro biomechanical experimental data reported in previous study [23, 24, 25, 26] (Figure 2), thereby validating the effectiveness of the intact L1–L5 model.

### 3.2 ROM of the Fixation Models

**FIGURE 3:**
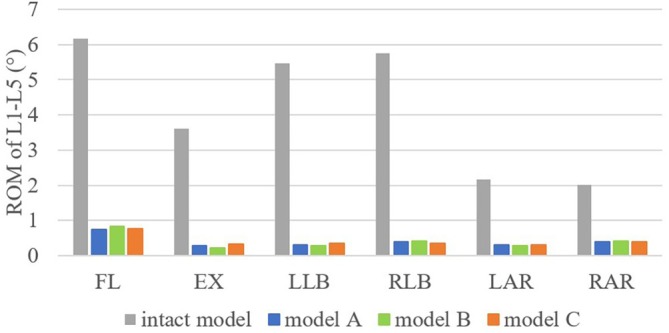
ROM of the intact model and the three models during FL, EX, LLB, RLB, LAR, and RAR. EX, extension; FL, flexion; LAR, left axial rotation; LLB, left lateral bending; RAR, right axial rotation; RLB, right lateral bending; ROM, Range of motion.

The ROMs of the postoperative models were presented in Figure 3. Notably, the ROMs of the three models were significantly restricted in all directions. Consequently, the prostheses provided good stability after implantation into the artificially excised L3 segment. The best stability of models A and C was similar, both observed during LAR (0.297° and 0.296°, respectively), while Model B had the best stability during EX (0.210°). The largest ROM among the three models was observed during FL in model B (0.884°). Overall, there were no significant differences in ROMs in the five directions: FL, LLB, RLB, LAR, and RAR among the three models. Compared with model C, the ROMs during EX decreased by 15.2% and 36.4% for models A and B, respectively.

### 3.3 von Mises Stress of the L2 Inferior Endplate and L4 Superior Endplate

**FIGURE 4:**
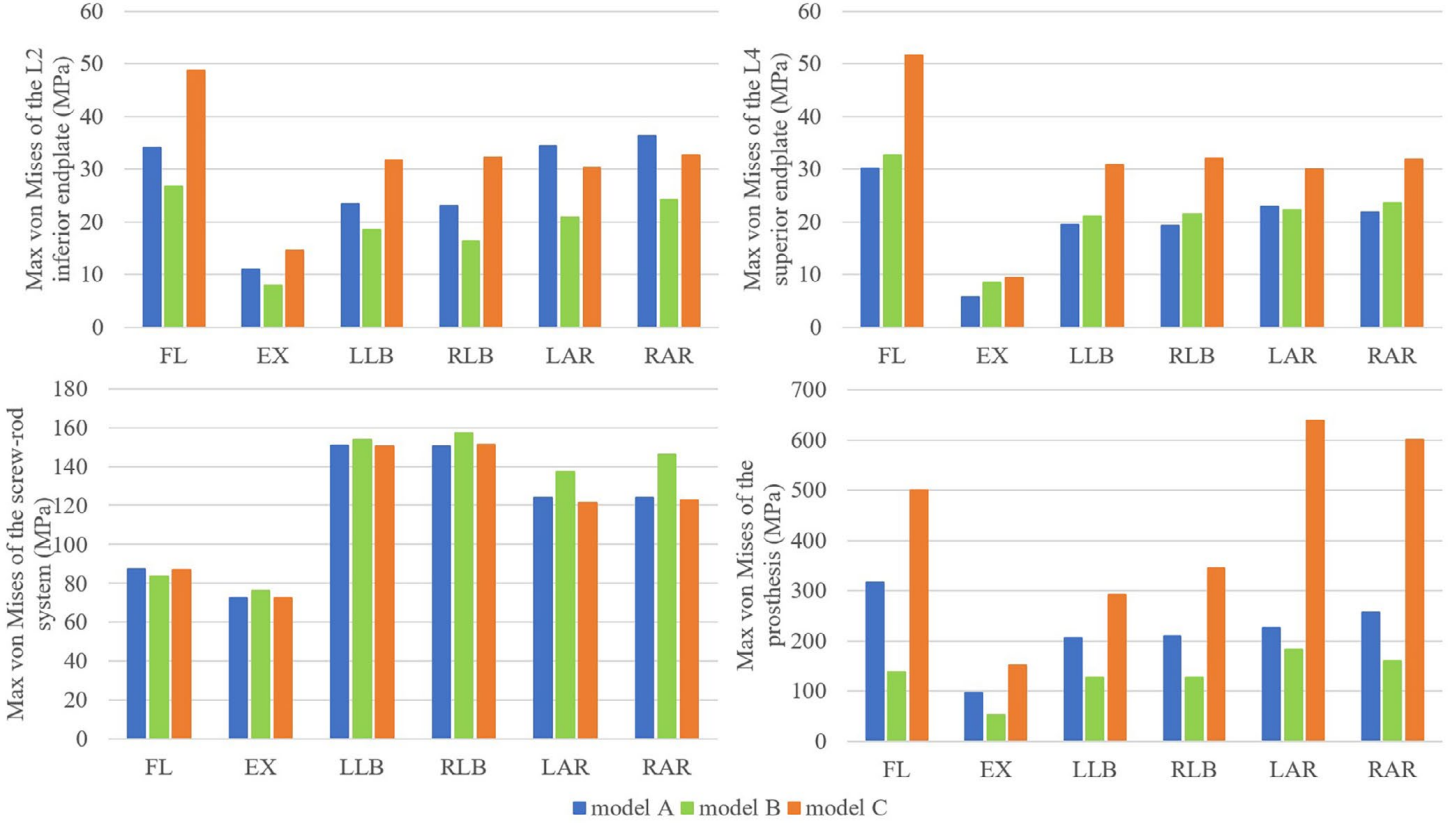
Maximum von Mises stress of the L2 inferior endplate, L4 superior endplate, screw‐rod system, and prosthesis of the three models during FL, EX, LLB, RLB, LAR, and RAR. EX, extension; FL, flexion; LAR, left axial rotation; LLB, left lateral bending; RAR, right axial rotation; RLB, right lateral bending.

**FIGURE 5:**
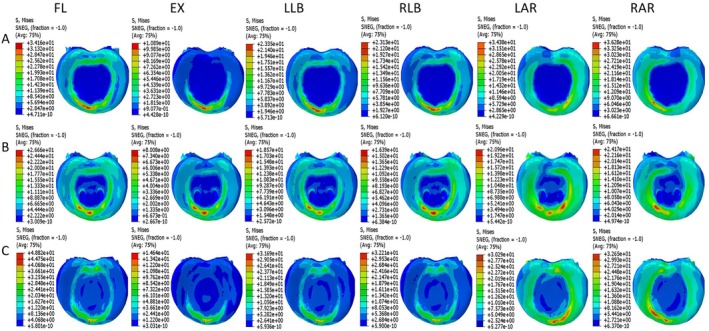
Von Mises stress of the L2 inferior endplate of the three models during FL, EX, LLB, RLB, LAR, and RAR. EX, extension; FL, flexion; LAR, left axial rotation; LLB, left lateral bending; RAR, right axial rotation; RLB, right lateral bending.

**FIGURE 6:**
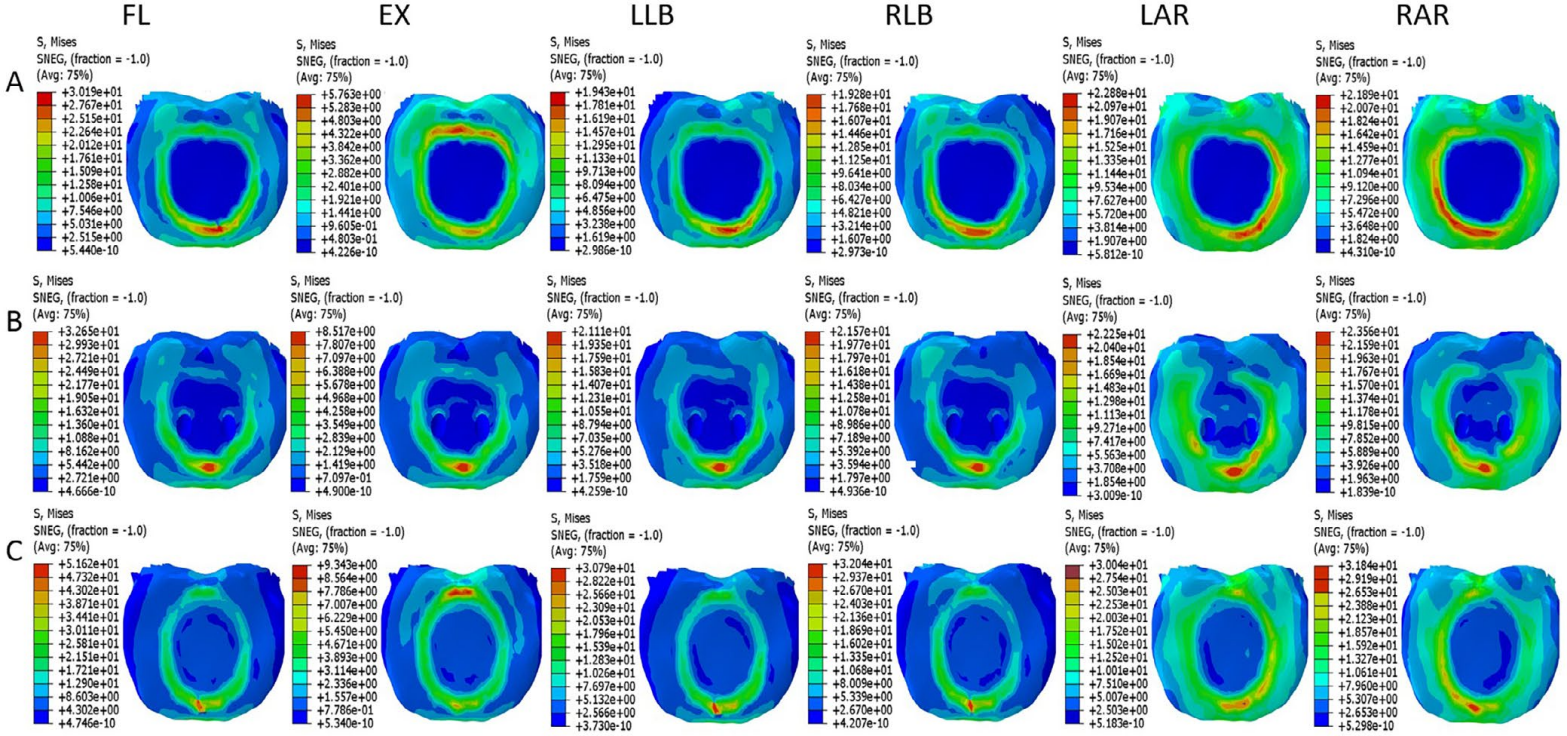
Von Mises stress of the L4 superior endplate of the three models during FL, EX, LLB, RLB, LAR and RAR. EX, extension; FL, flexion; LAR, left axial rotation; LLB, left lateral bending; RAR, right axial rotation; RLB, right lateral bending.

Regarding the endplates adjacent to the prosthesis, Figure 4 showed the maximum von Mises stress of the L2 inferior endplate and L4 superior endplate in the three models, and Figures 5 and 6 showed the specific magnitude and distribution of the stress. Among all directions of the three models, the stress of the L2 inferior endplate reached a maximum of 48.82 MPa in model C during FL. For each specific model, the largest stress in model A was observed during RAR (36.28 MPa) among all directions, while in models B and C, it was observed during FL, with values of 26.66 and 48.82 MPa, respectively. During FL, EX, LLR, and RLR, the maximum stress was largest in model C, followed by model A, and smallest in model B. Compared with model C, the most significant reductions in von Mises stress were observed in Models A and B, with decreases of 30.0% (34.16 vs. 48.82 MPa) during FL and 49.1% (16.39 vs. 32.21 MPa) during RLB, respectively. During LAR and RAR, the maximum stress was largest in model A, followed by model C, and smallest in model B; however, though the stress of the endplate in model A was larger than that in model C, there was almost no obvious stress concentration area on the L2 inferior endplate in contact with model A from the stress distribution condition, while apparent stress concentration areas were observed in model C.

Regarding the L4 superior endplate, the largest stress of the endplate among all directions in each model was also observed during FL, with the largest stress observed in model C, reaching 50.12 MPa. In all directions, the maximum stress of the L4 superior endplate was largest in model C, followed by model B, while that in model A was smallest. The most significant difference in stress was still observed during FL. Compared with model C, the stress in models A and B decreased by 41.5% (30.10 vs. 50.12 MPa) and 36.7% (32.65 vs. 50.12 MPa), respectively. During EX, the maximum stress of the endplate of model B decreased by 8.8% compared with model C, whereas in all other conditions, the stress of models A and B decreased by more than 20%.

### 3.4 von Mises Stress of the Screw‐Rod System

**FIGURE 7:**
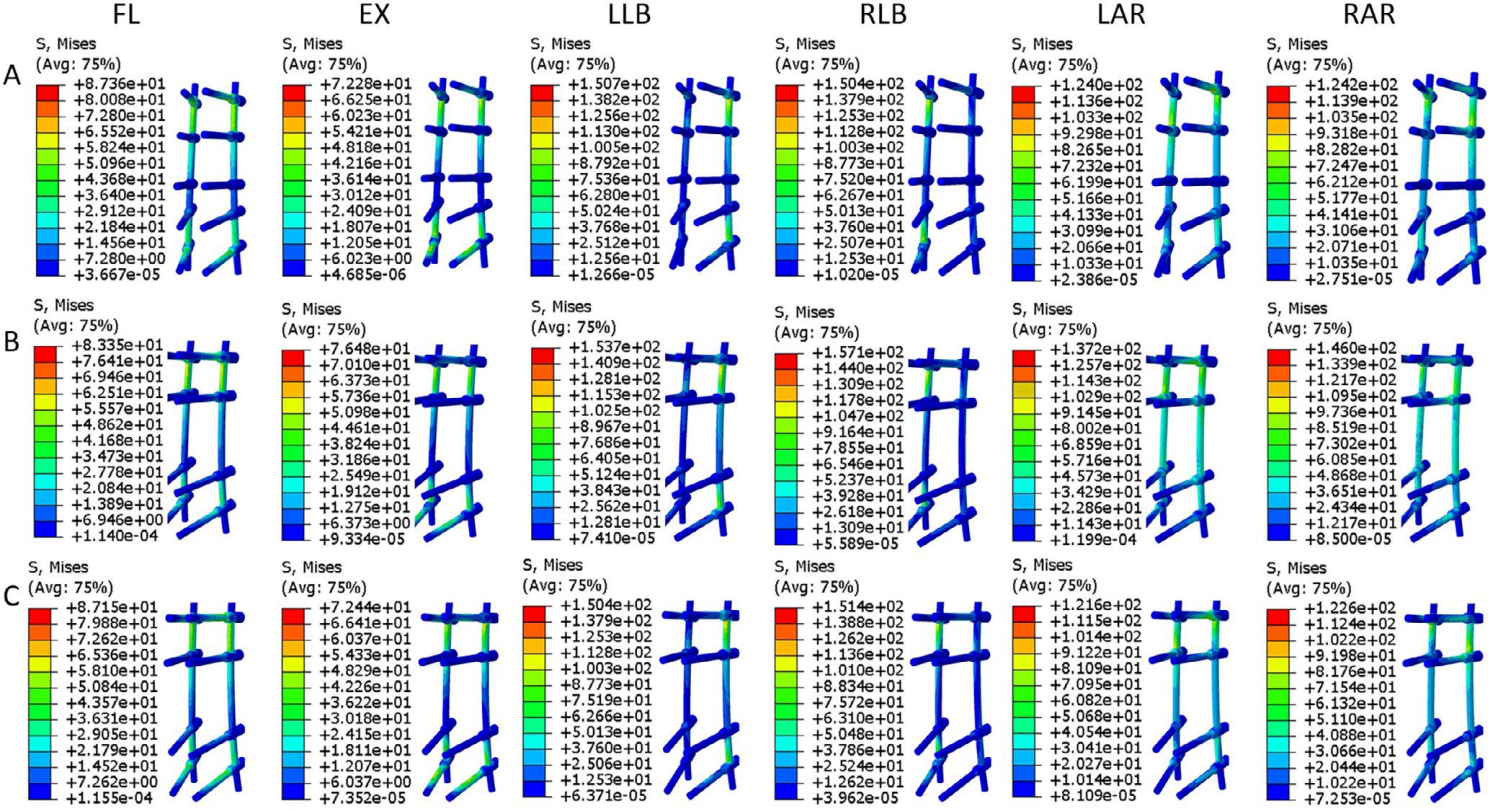
Von Mises stress of the screw‐rod system of the three models during FL, EX, LLB, RLB, LAR and RAR. EX, extension; FL, flexion; LAR, left axial rotation; LLB, left lateral bending; RAR, right axial rotation; RLB, right lateral bending.

In all three models, the maximum stress of the screw‐rod system was observed during LB, followed by AR (Figure 7). The largest stresses of models A, B, and C were 150.7 MPa (LLB), 157.1 MPa (RLB), and 151.4 MPa (RLB), respectively. In model B, the maximum stress of the screw‐rod system during AR was larger than that of models A and C, with increases of 10.6% and 12.8% during LAR (137.2 vs. 124 MPa, 137.2 vs. 121.6 MPa), and increases of 17.6% and 19.1% during RAR (146 vs. 124.2 MPa, 146 vs. 122.6 MPa), respectively. However, in the other four directions, the stresses of the screw‐rod system among three models were similar and did not differ substantially. The maximum stress of the three models was predominantly concentrated at the posterior rod in all directions.

### 3.5 von Mises Stress of the Different Prostheses

**FIGURE 8:**
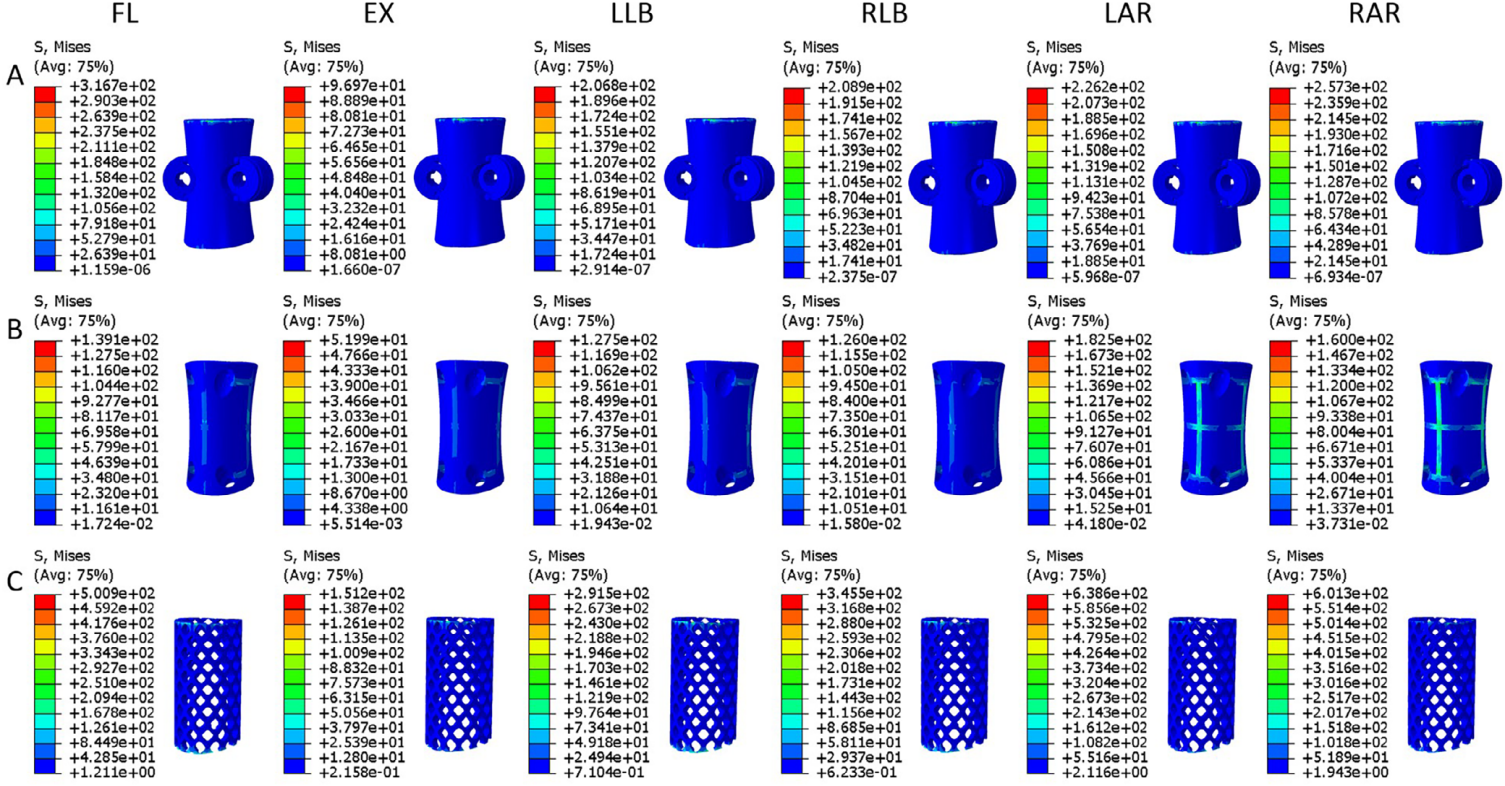
Von Mises stress of the prosthesis of the three models during FL, EX, LLB, RLB, LAR and RAR. EX, extension; FL, flexion; LAR, left axial rotation; LLB, left lateral bending; RAR, right axial rotation; RLB, right lateral bending.

Regarding anterior column reconstruction, the maximum stress of the three types of prosthesis varied significantly. As shown in Figure 8, the stress of the TMC was consistently much larger than that of the 3D‐printed prostheses in all directions. For the two 3D‐printed prostheses, the stress of the prosthesis in model B was consistently smaller than that in model A. The largest stress of the prosthesis was observed during LAR in model C, reaching as high as 638.6 MPa, while the smallest stress was observed during EX in model B, only 51.99 MPa. The largest stress of the prosthesis in model A was observed during FL (316.7 MPa), while in model B, it was observed during LAR (only 182.5 MPa). The maximum stress of the prosthesis in model C was 2.82 times larger than that in model A (638.6 vs. 226.2 MPa) during LAR. And during RAR, the maximum stress was 3.76 times larger than that in model B (601.3 vs. 160 MPa). These differences were the most significant among all six directions. Generally, the smaller the contact area between the prosthesis and the endplate, the larger the stress of the prosthesis. The results also supported this observation.

## 4 Discussion

Internal fixation failure is a common complication following TES, and achieving long‐term spinal stability remains a major clinical challenge [27]. Therefore, it is crucial to investigate the biomechanical alterations of the spine after TES [28]. This study investigated the biomechanical effects of three different types of prosthesis: model A (TES model with 3D‐printed prosthesis with artificial pedicle); model B (TES model with stand‐alone 3D‐printed prosthesis); and model C (TES model with TMC) in single‐segmental TES. The simulation results showed that the prosthesis used in anterior column reconstruction mainly affected the stress of adjacent endplates and the prosthesis itself, while the posterior fixation range had limited influence on overall ROM. The ROMs of three prosthesis implant models were significantly smaller than that of the intact model, with the maximum ROM of 0.884° far below the 5° threshold. However, during EX, the ROMs of 3D‐printed prostheses were significantly smaller than that of TMC. The stress of adjacent endplates in 3D‐printed prosthesis models was significantly lower than that of the TMC model in all directions, particularly during FL. The maximum stress of TMC was significantly larger than that of 3D‐printed prostheses in all directions, while the stand‐alone 3D‐printed prosthesis demonstrated the lowest maximum stress. The screw‐rod system stress of the three models remained far below the yield threshold, indicating adequate safety; however, stress concentration was primarily located at the posterior rod. Three postoperative models achieved good immediate stability and demonstrated favorable potential for intervertebral fusion.

### 4.1 Spinal Stability and ROM

Our findings demonstrated that all three prosthesis implant models achieved satisfactory immediate stability, with maximum ROM of 0.884°—far below the 5° threshold defined by the Food and Drug Administration for successful intervertebral fusion [10]. This finding aligns with previous finite element analyses [29, 30, 31], confirming that TES with posterior pedicle screw fixation and anterior column reconstruction can provide sufficient mechanical stability to promote fusion.

Notably, during EX, 3D‐printed prostheses showed significantly smaller ROMs than TMC (15.2% and 36.4% reduction for models A and B, respectively). This advantage likely stems from the improved endplate contact area achieved through personalized 3D‐printing design, which enhances load distribution and resistance to displacement. However, in other directions, prosthesis type had limited effects on overall ROM. This observation is consistent with Xu et al. [32], who reported that fixation range significantly impacts ROM, and Pflugmacher et al. [33], who demonstrated that posterior segment length affects overall fixation strength more than anterior implant selection. These results suggest that while 3D‐printed prostheses offer biomechanical advantages, surgical technique—particularly the extent of posterior fixation—remains the primary determinant of spinal stability. Additionally, this study used the lumbar spine as the experimental subject, incorporating the influence of normal physiological curvature of the spine on biomechanics. Different physiological curvatures may lead to mismatches between the prosthesis and vertebral body after vertebrectomy, affecting spinal stability. This is a common issue that spinal surgeons should consider.

### 4.2 Endplate Stress and Prosthesis Subsidence Risk

Prosthesis subsidence represents a critical complication following TES, reported in up to 63% of cases [34], often resulting from excessive endplate stress. Our analysis revealed that 3D‐printed prostheses significantly reduced adjacent endplate stress compared to TMC across all loading directions. Specifically, during FL, models A and B decreased L4 superior endplate stress by 41.5% and 36.7%, respectively. Several mechanisms explain this phenomenon. First, 3D‐printing technology enables personalized design with precise endplate matching, substantially increasing contact area and eliminating point‐contact stress concentrations inherent to TMC's sharp edges [2]. Second, the porous structure of 3D‐printed prostheses reduces elastic modulus, minimizing stress shielding and the “cutting effect” on vertebral endplates. Third, model A's artificial pedicle structure enhances stress distribution between the prosthesis and posterior fixation system, consistent with Colman et al. [35], who reported that artificial pedicle fixation can reduce adjacent cancellous bone subsidence by up to 50%. Therefore, the use of 3D‐printed prostheses is an effective strategy for reducing the risk of prosthesis subsidence [36, 37].

Model B demonstrated particularly impressive results, with L2 inferior endplate stress decreasing by 49.1% during FL. This design incorporates two screws into adjacent vertebrae, creating novel biomechanical interactions that prevent vertebral displacement while maintaining lower overall stiffness. To our knowledge, this represents the first biomechanical investigation of stand‐alone 3D‐printed prostheses in TES reconstruction, highlighting their potential to reduce subsidence risk without requiring posterior artificial pedicle fixation.

Importantly, the maximum endplate stress recorded (51.62 MPa) remained well within the reported cortical bone fracture strength range (90–200 MPa) [38], suggesting all models provide adequate safety margins against vertebral fracture.

### 4.3 Screw‐Rod System Stress and Fixation Failure

Screw loosening and rod fracture constitute major causes of internal fixation failure following TES, with rod fracture incidence reported as high as 40% [2, 39]. Our analysis revealed that the maximum screw‐rod system stress occurred in model B during RLB (157.1 MPa), which remains far below the 825 MPa yield threshold [37], indicating adequate safety for all reconstruction strategies. Stress concentration consistently occurred at the posterior rods across all models, correlating with clinical observations that rod fractures represent the most common mechanical complication [10, 39]. Interestingly, model B exhibited higher maximum stress than models A and C during AR, possibly because the stand‐alone design allows greater motion, distributing more stress to the posterior fixation. However, this increased stress did not approach critical thresholds and may actually represent favorable load sharing that could promote fusion.

Contrary to Wang et al. [31], who reported that artificial pedicle screws reduced posterior rod stress by 28.1% in short‐segment fixation, we did not observe significant stress reduction in model A compared to model C. This discrepancy may relate to differences in fixation length, as Xu et al. [32] demonstrated that 3D‐printed prostheses significantly reduce screw‐rod stress only when fixation extends from four to six segments. This represents an important area for future investigation.

### 4.4 Prosthesis Stress Distribution

The maximum stress observed in TMC (638.6 MPa during LAR) was 2.82 and 3.50 times higher than models A and B, respectively. Two reasons may explain this phenomenon. First, the elastic modulus of 3D‐printed prostheses is similar to that of human cortical bone, while much smaller than that of TMC, effectively reducing the stress shielding effect. Second, the design of 3D‐printed prostheses increases the contact area with the endplate, facilitating more uniform stress distribution across the prosthesis. However, significant stress concentration was observed at the limited contact points between TMC and the endplate.

Surprisingly, model B demonstrated significantly lower maximum prosthesis stress than model A across all directions (56.1% reduction during FL). We hypothesize that the four screws in model B's stand‐alone design share and transmit portions of the mechanical load, whereas model A's more rigid artificial pedicle fixation concentrates stress within the prosthesis structure. Additionally, model B's relatively higher mobility may allow more favorable stress distribution through micromotion, though this requires further biomechanical and clinical validation.

### 4.5 Limitations

This study has several limitations. First, the intact model was developed from a single CT dataset of a healthy volunteer, potentially limiting generalizability to the broader population and precluding absolute value determinations for stress and ROM. Second, the FEM did not incorporate paravertebral muscles or other surrounding soft tissues, which influence spinal mechanics in vivo. Consequently, we could not simulate muscle contraction effects or evaluate combined torque conditions under truly physiological loading scenarios. Third, material properties were simplified for computational efficiency, and we did not model pathological variations such as degeneration, ligament hypertrophy, disc dehydration, decreased disc height, or osteoporosis that commonly affect spinal tumor patients. Fourth, our results were restricted to single‐segmental TES; multi‐segmental reconstruction biomechanics may differ substantially. Finally, FEA represents a static biomechanical assessment that cannot fully replicate the dynamic, cyclic loading conditions of daily activities or long‐term biological processes such as bone remodeling and fusion. Despite these limitations, this study provides valuable insights for prosthesis selection in TES reconstruction. Our comparative analysis of three distinct reconstruction strategies, including the novel stand‐alone 3D‐printed prosthesis design, offers clinically relevant guidance.

### 4.6 Prospect

Future research should focus on: (1) cadaveric validation of these finite element findings; (2) prospective clinical studies tracking actual implant performance and subsidence rates; (3) dynamic finite element analyses incorporating muscle forces and cyclic loading; (4) patient‐specific modeling accounting for bone quality variations; and (5) long‐term follow‐up studies correlating predicted stress distributions with actual clinical outcomes. Such multi‐modal validation would substantially enhance the clinical applicability of biomechanical research in spinal reconstruction.

## 5 Conclusion

After single‐segmental TES, the approach of posterior fixation combined with anterior prosthesis reconstruction is effective in maintaining postoperative spinal stability. The differences of the stress of fixation systems are apparent when different types of prosthesis are implanted. Compared with TMC, 3D‐printed prostheses with larger contact area and smaller elastic modulus are demonstrated to exhibit superior biomechanical properties. Based on our results, the application of two types of 3D‐printed prosthesis could effectively reduce the risk of internal fixation failure and achieve better spinal stability.

## Author Contributions

**Jiasheng Chen:** writing – original draft, software, conceptualization, methodology. **Ben Wang:** writing – original draft, validation. **Yang Luo:** data curation, investigation. **Xiang Li:** data curation, formal analysis. **Yanchao Tang:** validation, investigation. **Zihe Li:** data curation, formal analysis, project administration. **Hua Zhou:** validation, investigation. **Panpan Hu:** investigation, validation. **Xiaoguang Liu:** writing – review and editing, validation. **Zhongjun Liu:** project administration, writing – review and editing, supervision. **Feng Wei:** writing – original draft, software, conceptualization, methodology.

## Funding

The authors received financial support from the National Key Research and Development Program of China (2023YFC3604400) and research grant of Peking University Third Hospital, Haidian District, Beijing, China (No. BYSYDL2023003).

## Ethics Statement

This study was approved by the Ethics Committee of Peking University Third Hospital (No. M2023797). The volunteer provided written informed consent. All clinical investigations had been conducted in accordance with the principles expressed in the Declaration of Helsinki.

## Consent

The authors have nothing to report.

## Conflicts of Interest

The authors declare no conflicts of interest.

## Data Availability

The data that support the findings of this study are available from the corresponding author upon reasonable request.
